# Preparation of LiFePO_4_ Powders by Ultrasonic Spray Drying Method and Their Memory Effect

**DOI:** 10.3390/ma14123193

**Published:** 2021-06-10

**Authors:** Tu Lan, Xiaolong Guo, De Li, Yong Chen

**Affiliations:** 1 State Key Laboratory on Marine Resource Utilization in South China Sea, Hainan Provincial Key Laboratory of Research on Utilization of Si-Zr-Ti Resources, School of Materials Science and Engineering, Hainan University, Haikou 570228, China; 18085204210017@hainanu.edu.cn; 2 Guangdong Key Laboratory for Hydrogen Energy Technologies, School of Materials Science and Hydrogen Energy, Foshan University, Foshan 528000, China

**Keywords:** LiFePO_4_, spray drying method, memory effect, cathode materials

## Abstract

The memory effect of lithium-ion batteries (LIBs) was first discovered in LiFePO_4_, but its origin and dependence are still not clear, which is essential for regulating the memory effect. In this paper, a home-made spray drying device was used to successfully synthesize LiFePO_4_ with an average particle size of about 1 μm, and we studied the influence of spray drying temperature on the memory effect of LiFePO_4_ in LIBs. The results showed that the increasing of spray drying temperature made the memory effect of LiFePO_4_ strengthen from 1.3 mV to 2.9 mV, while the capacity decreased by approximately 6%. The XRD refinement and FTIR spectra indicate that the enhancement of memory effect can be attributed to the increment of Li–Fe dislocations. This work reveals the dependence of memory effect of LiFePO_4_ on spray drying temperature, which will guide us to optimize the preparation process of electrode materials and improve the management system of LIBs.

## 1. Introduction

Due to energy shortage and environmental pollution, lithium-ion batteries (LIBs) have attracted enormous attention for their high energy density, long service life, and excellent safety performance [1,2,3,4]. With the development of science and technology, LIBs exhibit a lot of applications, which require superior performances [5,6,7]. As an important component of LIBs, the cathode material directly affects the electrochemical performance of LIBs [8,9,10,11]. Olivine-type LiFePO_4_, which has a high theoretical specific capacity of 170 mAh/g, excellent thermal stability, environmental friendliness and low price, is considered to be a promising cathode material for LIBs [12,13]. The research on LiFePO_4_ mainly focuses on electronic conductive coating [14,15], ion doping [16,17], particle size optimization [18,19], such as reducing the particle size of LiFePO_4_ to overcome weak ionic conductivity, using carbon coating on active particles to improve electronic conductivity.

LiFePO_4_ is usually synthesized by high-temperature solid-phase method, liquid-phase method, coprecipitation method, microwave heating and other methods [20,21,22,23]. High-temperature solid-state method is widely used and realizes industrial production due to its simple process, easy control of preparation conditions. However, the prepared electrode materials have the disadvantages of irregular particle shape, large grain size, unstable electrochemical performance, etc. Wet chemical methods, such as sol–gel method, hydrothermal method and coprecipitation method, can mix raw materials at molecular level with low temperature [24,25]. The prepared cathode materials have good conductivity, small particle size and uniform distribution, but high cost severely limits the output. The spray drying method, a method for atomizing the precursor solution into fine mist droplets, and then instantly drying them to solid particles in a high-temperature environment, has been widely used to prepare spherical micro powder in food, medicine, electronics, materials and other fields [26]. This method can achieve continuous production, and the prepared material particles have high purity and uniform and controllable size [27,28,29,30,31].

Eight years ago, Sasaki et al. [32] first discovered the memory effect of LiFePO_4_ in LIBs, which was also found in other two-phase materials later [33,34]. The memory effect refers to the fact that the battery memorizes the history of charge and discharge, and it can affect the battery performance, such as reducing the specific capacity and the service time [35]. As a voltage bump or step during the charging and discharging plateau, the memory effect can delay the two-phase transition, affect the estimation of the state of charge (SOC) and reduce the energy efficiency of LIBs [36,37]. In recent years, the memory effect of LIBs has been investigated from virous aspects, such as the relaxation time after phase transition and sintering temperature [37], particle size [36], ion doping [34], memory writing process [38], lithium excess [39], and oxygen vacancies [40].

In our previous work, the memory effect of LiFePO_4_ was obviously dependent on the relaxation time after the phase transition, of which the voltage bump was actually a delayed voltage overshooting [37], and it is also affected by the particle size of LiFePO_4_ [36]. Although the sintering temperature was proved to affect the memory effect of LiFePO_4_ [37], there is no report about the influence of spray drying temperature on the memory effect. In this work, a series of LiFePO_4_ samples were prepared by using home-made spray drying equipment, characterized by TGA analysis, SEM images, XRD refinement and FTIR spectra, in order to study the influence of spray drying temperature on the memory effect.

## 2. Materials and Methods

### 2.1. Preparation of LiFePO_4_ by Spray Drying Method

First, 0.036 mol LiH_2_PO_4_ (99.9%, Aladdin, Shanghai, China), 0.036 mol FeCl_2_▪4H_2_O (99.9%, Aladdin, Shanghai, China), 0.00108 mol LiOH▪H_2_O (99.9%, Aladdin, Shanghai, China), 15 mL hydrochloric acid (36–38%, Xilong Chemical, Guangzhou, China) and 0.85188 g sucrose (99.9%, Aladdin, Shanghai, China) were successively added into 50 mL deionized water, diluted to 200 mL and the precursor solution was obtained after thorough stirring. The precursor solution was atomized by the ultrasonic atomizer (402AI, Yuewell Company, Suzhou, China) at a frequency of 1.7 MHz, and then brought into a tube furnace (BTF-1100C-S, Anhui Bei Keke Equipment Technology Co., Ltd., Anhui, China) by 5% H_2_/Ar at various temperatures (200 °C, 250 °C, 300 °C and 350 °C, respectively) after the negative ion generator was turned on. Before this process, the air in the tube furnace was replaced with 5 L/min of 5% H_2_/Ar for 15 min. After spray drying, the LiFePO_4_ precursor powder was ground for 1 h and placed into the tube furnace. Under the 5% H_2_/Ar atmosphere, the furnace temperature was raised to 650 °C at a heating rate of 10 °C/min and kept for 8 h. The LiFePO_4_ samples were collected after naturally cooling to room temperature.

### 2.2. Characterization of Materials

D2 PHASER with filtered Cu Kα radiation, produced by Bruker company of Germany, was used to test the X-ray diffractometer (XRD, Bruker D8 advance, Bruker, Karlsruhe, Germany). The high-quality XRD patterns were collected by step scanning with the scanning range of 10° to 80° and a step width of 0.01° at room temperature. The Rietveld refinement was carried out by the General Structure Analysis System (GSAS 1.00, Regents of the University of California, CA, USA) with the EXPGUI interface [41]. The refinement process is as follows: the background and scale factor parameters are firstly determined; the scale factor is refined and 20 background coefficients are used for the Chebyshev polynomial function; the following instrumental/structural parameters, zero-shift, lattice parameters and profile parameters are refined. The thermal analysis was conducted on Q600SDT (TA Instruments, New Castle, DE, USA) at a heating rate of 10 °C min^−1^ from room temperature to 850 °C with an air flow of 20 mL/min. FTIR spectra were collected on PerkinElmer FTIR Spectrometer (FTIR, Perkin-Elmer Frontier, Perkin-ElmER, Waltham, MA, USA) with a resolution of 1 cm^−1^. Then, the morphology of as-prepared LiFePO_4_ precursor powders were characterized by scanning electron microscope (SEM, Phenom ProX, Phenom-World BV, Eindhoven, Netherlands).

### 2.3. Electrochemical Characterization

LiFePO_4_, acetylene black and binder PTFE were mixed with a mass ratio of 42.5:42.5:15 and rolled into a film. The film was cut into a disc with a diameter of 10 mm and pressed evenly on an aluminum mesh, dried at 80 °C for 12 h in a vacuum drying oven, and the cathode was prepared. The LiFePO_4_ film, lithium metal and Celgard 2400 microporous polypropylene film are positive electrode, negative electrode and separator respectively, and the electrolyte is 1 mol/L LiClO_4_/EC + DEC (volume ratio 1:1). The CR2025 button cell was assembled in a glove box and tested after rest for 12 h. The galvanostatic current charge/discharge test was carried out in the voltage range of 2.8 V to 4.0 V at 25 °C by the Hokuto Denko battery test system (HJ1001SD8, Hokuto Denko Corporation, Gifu, Japan).

## 3. Results and Discussion

**Figure 1:**
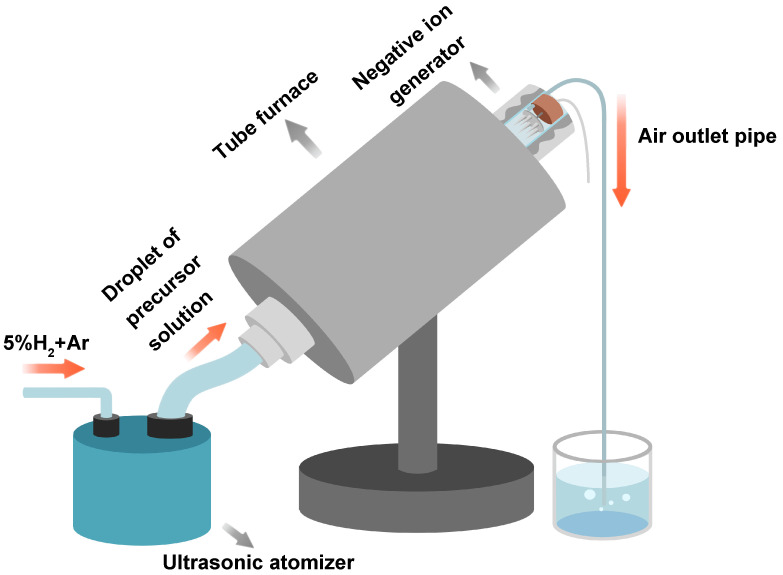
Schematic diagram of home-made spray drying device.

Figure 1 is a schematic diagram of a home-made spray drying device, which mainly consists of an ultrasonic atomizer, a tubular furnace, a negative ion generator and an air outlet pipe. Before starting the spray experiment, the airtightness of the device was confirmed to be in good condition. The air in the device was evacuated by introducing 5 L/min of 5% H_2_/Ar gas for 15 min. The nebulizer and negative ion generator were turned on at the same time, the precursor solution was atomized into fog droplets with an average particle diameter of 3.9 microns. The fog droplets were be carried into the inclined tubular furnace by 0.5 L/min of 5% H_2_/Ar gas. The fog droplets were quickly dried in contact with the high-temperature gas in the tube furnace to form solid particles. The gas in the tube furnace was partially ionized by the generator to generate negative ions, in which the solid particles tend to adsorb and deposit on the negative ion generator and the inner surface of the tube furnace. Then the gas was vented from the exhaust pipe. In this work, we adjusted the temperature in the tube furnace (200 °C, 250 °C, 300 °C, 350 °C) to study the influence of spray drying temperature on the memory effect of as-synthesized LiFePO_4_ samples.

**Figure 2:**
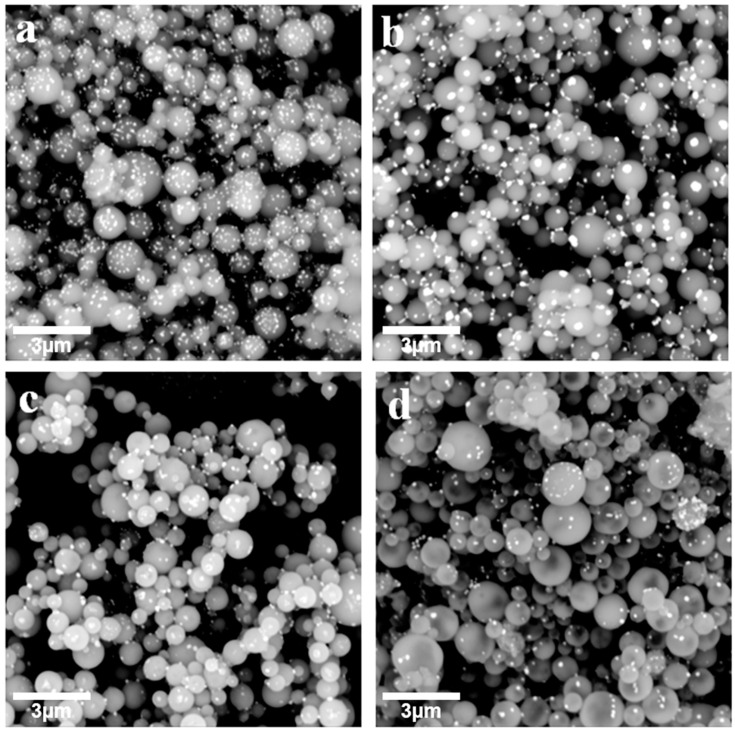
SEM photos of as-synthesized LiFePO_4_ precursor powder at spray drying temperature of (**a**) 200 °C, (**b**) 250 °C, (**c**) 300 °C and (**d**) 350 °C.

**Figure 3:**
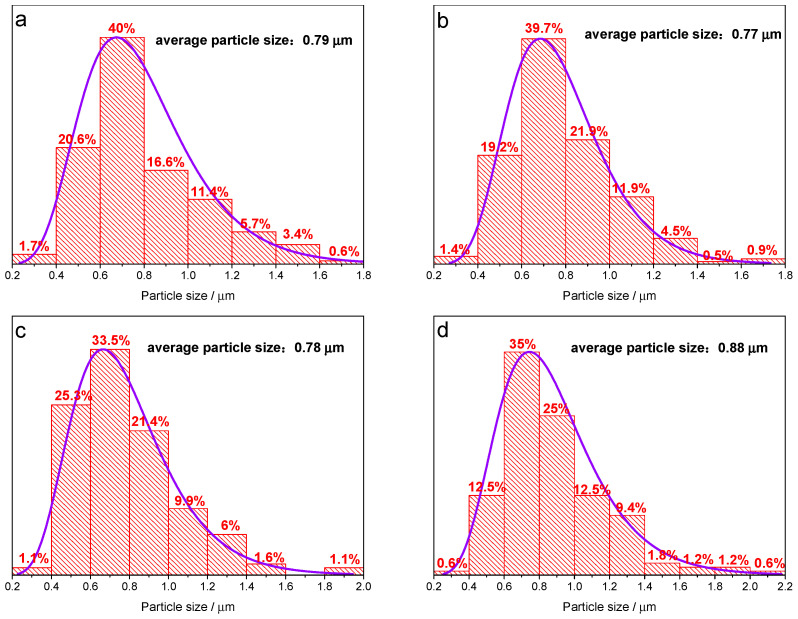
Particle size distribution of as-synthesized LiFePO_4_ precursor powder at spray drying temperature of (**a**) 200 °C, (**b**) 250 °C, (**c**) 300 °C and (**d**) 350 °C.

Figure 2 presents SEM photos of as-synthesized LiFePO_4_ precursor powder prepared at spray drying temperature of 200 °C, 250 °C, 300 °C and 350 °C, respectively. SEM images show that the LiFePO_4_ precursor particles are mainly spherical. Figure 3 shows the particle size analysis results of four temperatures, in which the particle size is mainly between 0.4 μm and 1.2 μm, accounting for more than 85%. The LiFePO_4_ precursor of 300 °C and 350 °C have a small number of particles with a diameter of more than 1.8 μm, while such particles are virtually absent for 200 °C and 250 °C. In addition, the average particle size for 200 °C, 250 °C and 300 °C is very close at 0.79 μm, 0.77 μm and 0.78 μm, respectively, while the average particle size for 350 °C is slightly larger as 0.88 μm. The precursor particle size in this work is concentrated below 1 micron, while the precursor particle size prepared by Yu F et al. is far greater than 1 micron [37,42]. In fact, the ultrasonic atomization method that we used can produce a smaller particle size, resulting in better electrochemical performance [43,44].

**Figure 4:**
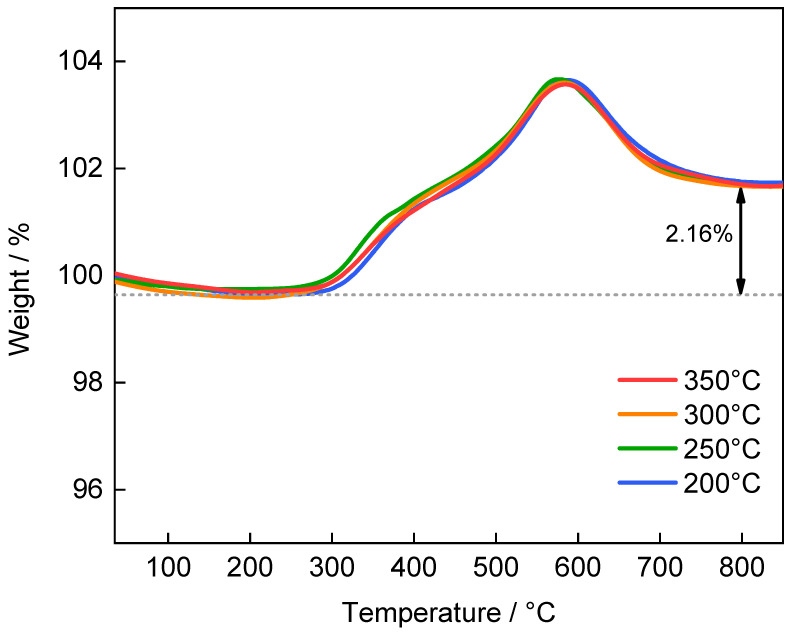
TGA curve of LiFePO_4_ samples prepared at spray drying temperature of 200 °C, 250 °C, 300 °C and 350 °C.

As shown in Figure 4, the thermogravimetric analysis (TGA) was carried out for LiFePO_4_ samples prepared at different spray drying temperatures. All samples exhibit very similar TGA curves, where the samples were heated from room temperature to 850 °C at a rate of 10 °C/min with an air flow of 70 mL/min. The weight loss below 310 °C can be attributed to the crystal water, which is about 0.36%. When the temperature rises to 310 °C, the LiFePO_4_ samples began to be oxidized to Li_3_Fe_2_(PO_4_)_3_ and Fe_2_O_3_, thus the weight increases by 5.07%, theoretically, based on the following reaction formula [45,46]:

(1) $$LiFePO_{4}+\frac{1}{4}O_{2}=\frac{1}{3}Li_{3}Fe_{2}{\left(PO_{4}\right)}_{3}+\frac{1}{6}Fe_{2}O_{3}$$

At about 580 °C, the carbon in the samples is oxidized into CO_2_ with a weight loss. Therefore, the total weight has increased by 1.8%, and the carbon content in the sample should be 2.91%. The carbon comes from sucrose in the precursor solution, which is used to improve the conductivity of LiFePO_4_. As we expected, the TGA result indicates that the LiFePO_4_ sample has standard thermal stability in air.

**Figure 5:**
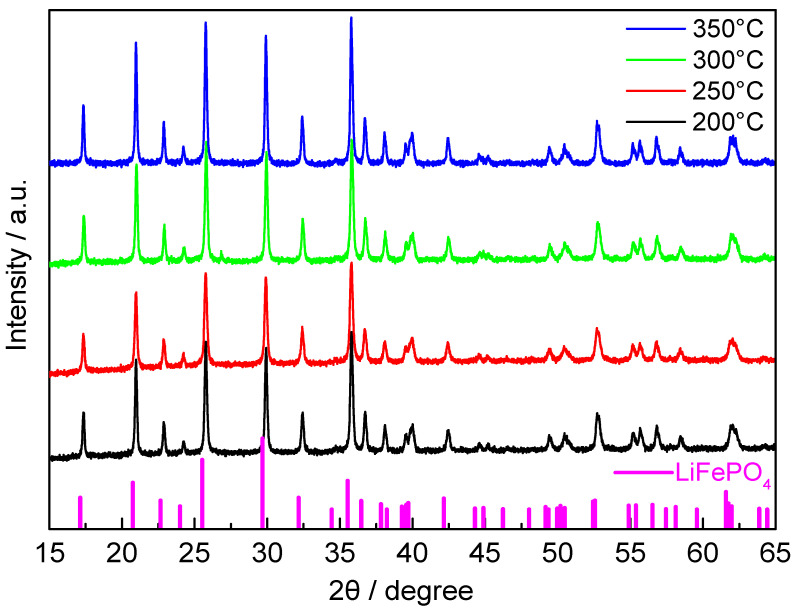
XRD patterns of LiFePO_4_ samples prepared at spray drying temperature of 200 °C, 250 °C, 300 °C and 350 °C.

**Figure 6:**
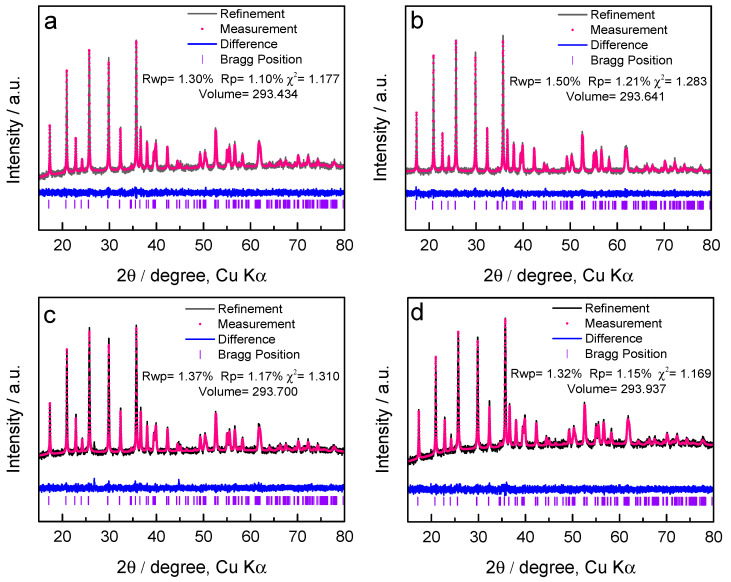
XRD spectra of LiFePO_4_ samples prepared at spray drying temperature of (**a**) 200 °C, (**b**) 250 °C, (**c**) 300 °C and (**d**) 350 °C, as well as Rietveld refinement of Pnma. The black line, the red circle and the blue line correspond to the observed pattern, the calculated diffraction pattern and the difference pattern.

As shown in Figure 5, the XRD patterns of all LiFePO_4_ samples are consistent with that of olivine LiFePO_4_ (PDF card number: 81-1173), indicating no impurity phase. All XRD data were analyzed by Rietveld refinement with General Structure Analysis System (GSAS) software [41], which is an important method to understand the crystal structure, cell parameters and other information of crystal materials. Figure 6a–d shows the refinement results calculated from Pnma phase group of LiFePO_4_ collected at 200 °C, 250 °C, 300 °C and 350 °C during the spray drying process. The black line, red circle and blue line correspond to the observed pattern, the calculated diffraction pattern and the difference pattern, respectively. There are no sharp peaks at the Bragg position of the blue difference curve, indicating a very successful fit. In addition, the Rietveld refinement results provide excellent fits based on the Rwp, Rp and χ2 fitting factors, and they are concentrated in very small ranges of 1.3% to 1.5%, 1.1% to 1.2% and 1.1 to 1.3, respectively.

The cell volume of LiFePO_4_ samples of 200 °C, 250 °C, 300 °C and 350 °C is 293.434 Å^3^, 293.641 Å^3^, 293.7 Å^3^ and 293.937 Å^3^, respectively. By increasing the spray drying temperature, the unit cell volume of LiFePO_4_ increases gradually, which is caused by the disorder of crystal structure. When Fe^2+^ ions in M2 position move to M1 position to replace Li ions, this Li–Fe dislocation will destroy the most stable structure of LiFePO_4_, resulting in distorted structure with larger cell volume. In fact, the Li–Fe dislocations are the most favorable defect in LiFePO_4_ and have the lowest formation energy [47], in which the Fe^2+^ ions will expand the unit cell along a and c, due to having a larger size than Li^+^ ions [48,49]. However, they barely affect the unit cell along b, for there is more channel space to accommodate the Fe^2+^ ions. Consequently, the disordered Fe^2+^ ions will block Li^+^ ions in the (101) channels that are for Li^+^ ions deintercalation in LiFePO_4_ [50,51]. Therefore, the Li–Fe dislocations should be dependent on the spray drying temperature.

**Figure 7:**
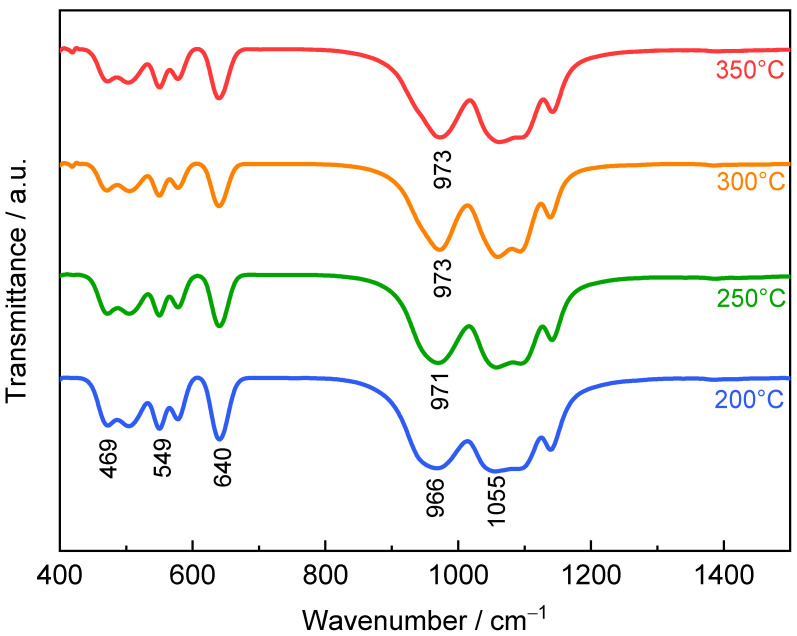
FTIR spectra of LiFePO_4_ samples prepared at spray drying temperature of 200 °C, 250 °C, 300 °C and 350 °C.

In Figure 7, the peaks of FTIR spectra locate at 469, 549, 640, 966, and 1055 cm^−1^ for LiFePO_4_ samples [52]. The peak at 463 cm^−1^ is due to the bending harmonics of O–P–O and O=P–O groups. The peaks located at 547 and 638 cm^−1^ are assigned to the stretching vibrations of the P–O–P group and the peak at 966 cm^−1^ corresponds to P–O–P bending modes. The band observed at 1043 cm^−1^ corresponds to vibration of (PO_4_)^3−^ link metal ions [53,54]. As the spray drying temperature increases, the blue shift of the peak at 966 cm^−1^ is correlated to the Li–Fe antisite defects [55,56], thus suggesting that the increase in spray drying temperature will increase dislocations of LiFePO_4_. This is consistent with the result of the XRD refinement.

**Figure 8:**
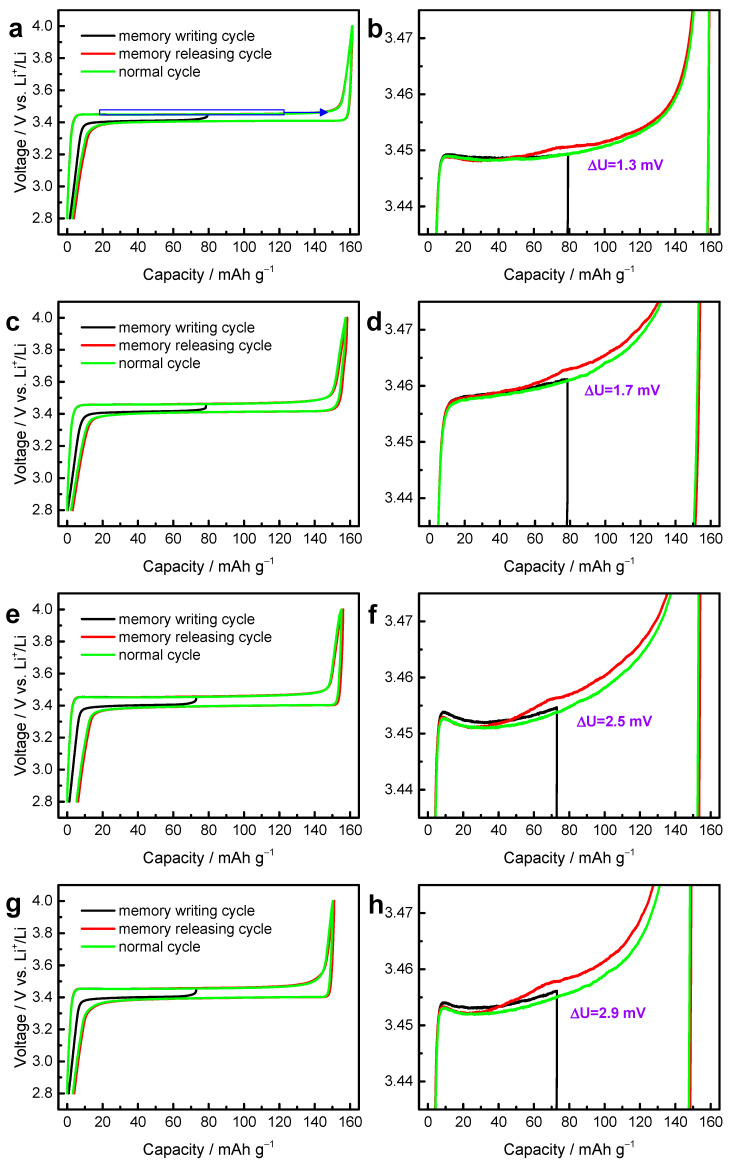
Determination of the memory effect during charging for LiFePO_4_. For memory effect during charging, the memory-writing cycle was a half-charge from 2.8 V and a discharge to 2.8 V (black); the memory-releasing cycle (red) and normal cycle (green) were a full charge–discharge from 2.8 V to 4.0 V. The memory effect is shown for LiFePO_4_ samples prepared at spray drying temperatures of (**a**,**b**) 200 °C, (**c**,**d**) 250 °C, (**e**,**f**) 300 °C and (**g**,**h**) 350 °C during charging, as well as (**b**,**d**,**f**,**h**) enlarged view of (**a**,**c**,**e**,**g**), respectively. Here, the current rate was 0.1C.

As shown in Figure 8a,c,e,g, LiFePO_4_ samples prepared at spray drying temperatures of 200 °C, 250 °C, 300 °C and 350 °C have specific capacity of 161.15 mAh/g, 157.4 mAh/g, 155 mAh/g and 151 mAh/g, which decreases as the spray drying temperature increases from 200 °C to 350 °C. Their memory effect is enhanced after increasing the spray drying temperature, as the ∆U, the potential gap between memory-releasing cycle and memory-writing cycle, is 1.3 mV, 1.7 mV, 2.5 mV and 2.9 mV for 200 °C, 250 °C, 300 °C and 350 °C, respectively. Combining the results of FTIR and XRD refinement, the reason that the memory effect of LiFePO_4_ increases with the spray drying temperature can be attributed to the increment of Li–Fe dislocations. In olivine LiFePO_4_, Li–Fe dislocations can block the [010] channel of Li-ion migration [57,58], which was proved by advanced electron microscopy, neutron diffraction (or X-ray diffraction) and theoretical calculations [39,51,59], so the lower specific capacity of LiFePO_4_ may be due to the increased Li–Fe dislocations, consistent with previously reports [25,60].

Except for the spray drying temperature, the memory effect of LiFePO_4_ has been studied by controlling the relaxation time, the voltage overshooting, the sintering temperature, the particle size, the lithium excess, etc., in previous investigations. As to the relaxation time [37], the memory effect is significantly dependent on the relaxation time after phase transition, and a rest of 20 h was added into the memory writing process to enhance the memory effect, while we also observed the evident memory effect without a rest in the memory-writing cycle for this work. As to the voltage overshooting [37], the voltage bump of memory effect is considered as a delayed voltage overshooting, which is overlaid at the edge of stepped (dis)charging plateau, while the voltage bump is small compared with the voltage step owing to the low sintering temperature of 650 °C in this work. As to the sintering temperature [37], the memory effect is noticeable for the high temperature of 800 °C, especially for the voltage bump at the step edge, while the increasing of spray drying temperature strengthened the memory effect of LiFePO_4_ in this work, so the high temperature in the synthesis process can lead to the strong memory effect. As to the particle size [36], the memory effect of micro LiFePO_4_ is stronger than that of nano LiFePO_4_, which can be attributed to the fact that the phase transition of micro particles is slower than that of nano particles, while the different spray drying temperature can also affect the particle size in this work, and the LiFePO_4_ sample prepared at a high spray drying temperature of 350 °C exhibits an evident memory effect, consistent with the previous work. As to the lithium excess [39], Kyu et al. studied the effect of excessive Li on the memory effect of LiFePO_4_, and the results showed that in the case of excessive Li, the memory effect of LiFePO_4_ was significantly reduced, due to the presence of Li_Fe_ and the absence of Fe_Li_ in lithium-excess olivine LiFePO_4_; similarly, the spray drying temperature affects the memory effect of LiFePO_4_ through changing the Li–Fe anti-site defects in this work.

## 4. Conclusions

In this work, we set up a convenient home-made spray drying piece of equipment, prepared a series of LiFePO_4_ with different spray drying temperatures, and studied their electrochemical performance in lithium-ion batteries. As the spray drying temperature varies from 200 °C to 350 °C, the memory effect of LiFePO_4_ was enhanced from 1.3 mV to 2.9 mV, and the specific capacity was reduced from 161 mAh/g to 151 mAh/g. XRD refinement and FTIR analysis show that the Li–Fe dislocations increase with the spray drying temperature in LiFePO_4_ samples. The defect of Li–Fe anti-site blocked some [010] channels of LiFePO_4_ structure to retard the Li-ion migration, resulting in the memory effect. Our results show that the spray drying temperature has a significant impact on the memory effect and specific capacity of electrode materials, which can be adopted to improve and optimize electrode materials.

## Data Availability

The data presented in this study are available on request from the corresponding author after obtaining permission from an authorized person.
